# Case Report: Equine allogeneic umbilical cord blood mesenchymal stromal cells (CB-MSC) as adjunctive therapy in a foal with septic arthritis and osteomyelitis

**DOI:** 10.3389/fvets.2026.1734585

**Published:** 2026-03-04

**Authors:** Marta Horna, Alejandro Merchán Muñoz, Laurie Goodrich, Lynn Pezzanite, Steven Dow, Thomas R. Koch, Amir H. Alizadeh, Sahar Mehrpouyan, Judith Koenig

**Affiliations:** 1 Department of Clinical Studies, Ontario Veterinary College, University of Guelph, Guelph, ON, Canada; 2 Department of Clinical Sciences, Colorado State University, College of Veterinary Medicine and Biomedical Sciences, Fort Collins, CO, United States; 3 Department of Biomedical Sciences, Ontario Veterinary College, University of Guelph, Guelph, ON, Canada; 4 eQcell Inc., University of Guelph, Guelph, ON, Canada

**Keywords:** bone lysis, hematogenous spread, neonate, pathologic fracture olecranon, septic elbow

## Abstract

Mesenchymal stromal cells (MSCs) are recognized for their potent anti-inflammatory, antibacterial, and immunomodulatory properties, making them a promising therapeutic option for combating antibiotic resistance and biofilm-associated infections. This report describes the successful treatment of septic arthritis and osteomyelitis in a foal using equine allogeneic cord blood-derived MSCs (CB-MSCs) in combination with antibiotic therapy. An 8-day-old Thoroughbred filly initially presented with septic arthritis of the right tibiotarsal joint, pneumonia, and omphalophlebitis/arteritis. Subsequently, the filly developed septic arthritis of the left elbow joint, epiphysitis, and osteomyelitis of the ulna, which progressed to an aggressive pathological fracture. Chloramphenicol was instituted based on the bacterial culture and susceptibility. Due to limited clinical and cytological improvement following needle lavage, arthroscopic lavage, and intra-articular antibiotic administration, the left elbow joint and fracture site were treated three times with 15 million TLR3-activated CB-MSCs in combination with meropenem (7.25 mg/kg IA). Additionally, the filly received twice systemic treatment with non-activated CB-MSCs (1 million cells/kg IV). The treatment resulted in complete resolution of both septic arthritis and osteomyelitis. At a 12-month follow-up, the filly remained sound, and radiographic re-evaluation showed significant remodeling of the ulna. This case describes the successful use of equine allogeneic cord blood–derived mesenchymal stromal cells (CB-MSCs), administered locally and systemically in combination with antibiotic therapy, to manage a refractory intra-synovial and osseous septic process in a foal. The use of TLR3-activated CB-MSCs may have supported antimicrobial treatment, highlighting the potential antimicrobial, anti-inflammatory, and immunomodulatory properties of CB-MSCs.

## 1 Introduction

The septic arthritis–osteomyelitis disease complex (SAPO), also referred to as hematogenous septic arthritis, physitis, and osteomyelitis, is an infectious orthopedic condition of foals that can result in severe systemic illness and may be fatal (1–3). The condition most commonly occurs in neonatal foals as a complication of sepsis; however, foals up to 7 months of age may also be affected (4).

SAPO has been reported to affect up to 1% of foals, with concurrent osseous involvement identified in approximately 26 to 78% of cases (1). Septic arthritis accompanied by physeal, epiphyseal, or metaphyseal osteomyelitis is consistently associated with a poorer prognosis, particularly when epiphyseal lesions or multiple joints are involved. Delayed diagnosis or inadequate treatment may contribute to disease progression, increasing the risk of persistent infection, systemic compromise, and mortality. In severe cases, euthanasia may be considered when pain, recumbency, or inability to nurse cannot be adequately managed. Survivors may develop long-term sequelae such as osteochondrosis, osteoarthritis, and reduced athletic performance (1–4).

Septic arthritis, particularly when complicated by osteomyelitis, is considered an orthopedic emergency requiring prompt and aggressive intervention. Current standard-of-care treatment includes systemic broad-spectrum antimicrobial therapy, joint lavage and debridement, and, in selected cases, intra-articular antimicrobial administration (5, 6). Management in foals is further complicated by the frequent presence of concurrent systemic conditions, such as failure of passive transfer and sepsis-associated organ dysfunction, which may influence treatment response and outcome (5).

Osteomyelitis is often diagnosed later in the disease course, as radiographic changes may lag behind clinical infection. Once bacterial colonization of the metaphysis or physis occurs, the associated inflammatory response can lead to ischemia and bone necrosis. Necrotic tissue may limit host immune activity and reduce penetration of systemically administered antimicrobials, thereby complicating infection control (5). Consequently, local therapeutic strategies, including surgical debridement and targeted antimicrobial delivery, are commonly incorporated into treatment protocols for SAPO (1, 7).

Antimicrobial resistance (AMR) represents an increasing concern in both human and veterinary medicine. Historically, Gram-negative organisms have been most frequently isolated in cases of neonatal septic arthritis, while Gram-positive organisms accounted for approximately one-third of isolates, primarily from blood cultures (5). More recent studies, however, have reported an increased frequency of Gram-positive bacterial isolation from joint and blood cultures in foals with septic arthritis (1, 8–12). In parallel, multidrug resistance has been documented in both Gram-positive and Gram-negative organisms, with up to 34% of Gram-negative isolates reported as multidrug-resistant in some studies (4, 13). These trends underscore the need to evaluate adjunctive and alternative therapeutic strategies while maintaining antimicrobial stewardship.

Mesenchymal stromal cells (MSCs) have been investigated in experimental and preclinical settings for their anti-inflammatory and immunomodulatory properties, as well as for their potential antimicrobial effects (14–17). *In vitro* and animal model studies suggest that MSCs may exert direct antimicrobial activity, including interference with bacterial biofilm formation, and may support the activity of concurrent antimicrobial therapy (14–16, 18). These findings, however, are largely derived from laboratory-based or non-equine studies, and their clinical relevance in foals with naturally occurring infections has not been established.

MSCs have also been shown in experimental models to influence host immune responses through paracrine signaling, cell–cell interactions, and secretion of bioactive molecules that may affect immune cell recruitment and activation (14–16, 19). While these immunomodulatory effects may be beneficial in inflammatory conditions, their role in the treatment of septic orthopedic disease remains investigational.

*In vitro* preconditioning (also termed priming or licensing) of MSCs using Toll-like receptor or NOD-like receptor ligands has been shown in experimental studies to alter MSC behavior, including changes in migratory capacity and modulation of immune responses (14, 15, 20). Evidence from rodent and canine models suggests that exposure to proinflammatory stimuli may shift MSCs toward phenotypes with altered immunomodulatory properties (14, 20–24). However, data supporting the safety, efficacy, and clinical utility of activated MSCs in equine orthopedic infectious disease remain limited.

This case report describes the adjunctive use of TLR3-activated and non-activated allogeneic equine umbilical cord blood–derived mesenchymal stromal cells (CB-MSCs) alongside conventional antimicrobial therapy in a foal with septic arthritis and osteomyelitis. This report focuses on clinical observations and feasibility and is not intended to establish efficacy or recommend routine clinical use.

## 2 Case description

### 2.1 Clinical history

An 8-day-old Thoroughbred filly was presented for evaluation and treatment of septic arthritis of the right tibiotarsal joint, pneumonia, and omphalophlebitis/arteritis. Initial management included surgical resection of the infected umbilical structures and repeated needle through-and-through lavage of the right tibiotarsal joint.

Synovial fluid culture from the tibiotarsal joint yielded *Escherichia coli*, which was susceptible to amikacin, ceftiofur, chloramphenicol, enrofloxacin, gentamicin, and tetracycline. Culture of the excised umbilical remnants identified three bacterial species: (1) *Streptococcus equi* subsp. *zooepidemicus*—susceptible to ampicillin, ceftiofur, chloramphenicol, erythromycin, rifampin, and trimethoprim-sulfamethoxazole (TMS); (2) *Enterococcus faecalis—*susceptible to ampicillin, chloramphenicol, erythromycin, penicillin, and tetracycline; (3) *Proteus vulgaris*—susceptible to amikacin, ceftiofur, chloramphenicol, enrofloxacin, gentamicin, and TMS.

Initial therapy consisted of systemic antimicrobial treatment with amikacin (25 mg/kg IV, q24h) and penicillin (22,000 IU/kg IV, q6h) administered for 6 days. Non-steroidal anti-inflammatory therapy included flunixin meglumine at 1.1 mg/kg IV q12h for 8 days, followed by 0.5 mg/kg IV q12h for an additional 14 days. Gastroprotective therapy consisted of omeprazole (4 mg/kg PO, q24h) and sucralfate (20 mg/kg PO, q8h) for 23 days. In addition, intra-articular administration of amikacin (4 mg/kg IA) was performed for 4 days. The filly responded well to treatment, with resolution of septic arthritis in the right tibiotarsal joint and satisfactory healing of the laparotomy incision.

However, on day 5 of hospitalization, the filly developed acute left forelimb lameness (4 out of 5 AAEP scale), accompanied by soft tissue swelling around the left elbow.

### 2.2 Diagnostic finding and interpretation

**Figure 1:**
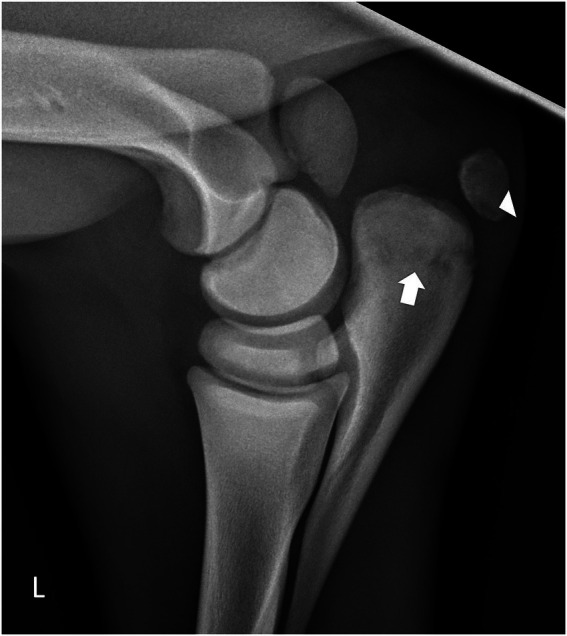
Mediolateral radiographic projection of the left radiohumerocubital joint. A radiolucent defect is visible within the proximal ulnar apophysis (white arrowhead). Additional radiolucent regions are evident in the proximal aspect of the ulna (white arrow), consistent with early osteomyelitis.

On day 6 of hospitalization, ultrasonographic examination of the left elbow demonstrated mild periarticular soft tissue edema without evidence of increased synovial effusion. Radiographic evaluation of the left radiohumeral (elbow) joint identified an irregular, linear radiolucency extending through the metaphysis of the proximal olecranon, along with a focal radiolucent region at the distal margin of the proximal olecranon apophysis (Figure 1). These imaging findings were considered most consistent with osteomyelitis complicated by a secondary pathologic fracture. However, alternative etiologies, including primary mechanical failure, bone fragility associated with systemic illness, or delayed detection of osseous involvement, could not be excluded.

By day 7 of hospitalization, the filly’s left forelimb lameness had progressed (4–5 out of 5 AAEP scale). Clinical examination revealed moderate synovial effusion of the left elbow joint. Synovial fluid analysis was consistent with a suppurative inflammatory process, characterized by a total nucleated cell count (TNCC) of 25,000 cells/μL, a total protein concentration of 40 g/L, and a neutrophil proportion of 88%. Consequently, systemic antimicrobial therapy was transitioned to chloramphenicol (50 mg/kg PO q6h) on day 7, and penicillin and amikacin were discontinued.

### 2.3 Treatment and outcome

Initial treatment of the left radiohumeral (elbow) joint consisted of through-and-through needle lavage, followed by intra-articular administration of meropenem (7.25 mg/kg IA) on day 7 of hospitalization. This protocol was repeated on days 9, 10, 12, and 14. Additionally, 15 million Toll-like receptor 3 (TLR3)–activated umbilical cord–derived mesenchymal stromal cells (CB-MSCs), prepared as previously described by Koch et al. (25), and Luque et al. (26), were administered both intra-articularly and directly into the pathological fracture site under ultrasonographic guidance on days 10, 12, and 14. In brief, MSCs were harvested from the umbilical vein of foals from Thoroughbred broodmares of various ages, only if the mares were healthy at the time of foaling and had normal complete blood counts and a negative Coggins test. The nucleated cell (NC) fraction was isolated from cord blood using red blood cell (RBC) lysis as previously described (27). The cells were transferred to a 1-layer Corning CellStack (Cell Bind) culture chamber and incubated at 38 °C in a humidified atmosphere containing 5% CO₂. After 12–18 h, the medium was replaced to remove non-adherent cells. Media changes were performed every 2–3 days until colonies of spindle-shaped fibroblast-like cells, characteristic of MSCs, were observed. Cultures were monitored daily for contamination and colony formation. When cell confluence reached 60–80%, sub-culturing was performed using TrypLE Express (Thermo Fisher). Cells were detached, split 1:6, and seeded in Corning CellStack chambers. Cells were expanded for 3 passages and frozen into preliminary cell banks. Thawed cells from 5 donors were pooled and expanded for clinical application until passage five (P5) and cryopreserved into clinical vials. A proprietary activation method was used to activate the MSCs by stimulating Toll-like receptor 3 (TLR3) for the 3 dosages administered locally. Non-activated CB-MSCs were used for the 2 IV infusions. The final CB-MSC product consisted of a pooled preparation of five independent CB-MSC cultures, each derived from a different equine cord blood donor. The same pooled CB-MSC product, derived from the same five donors, was used for all administered doses. All donor animals were Thoroughbred foals and of the 5 donors 3 were colts and 2 were fillies. Representative samples of the final CB-MSC product were submitted to the Animal Health Lab (University of Guelph) for antigen PCR testing and were negative for Equine Pegivirus, Equine Parvovirus, Hepacivirus, Theiler’s Disease Associated Virus, West Nile Virus, Equine Eastern Encephalitis Virus, Equine Herpes Virus-1, and Equine Viral arteritis virus. At the time of cryopreservation, cell culture samples were also tested for sterility by bacterial culture and endotoxin testing.

**Figure 2:**
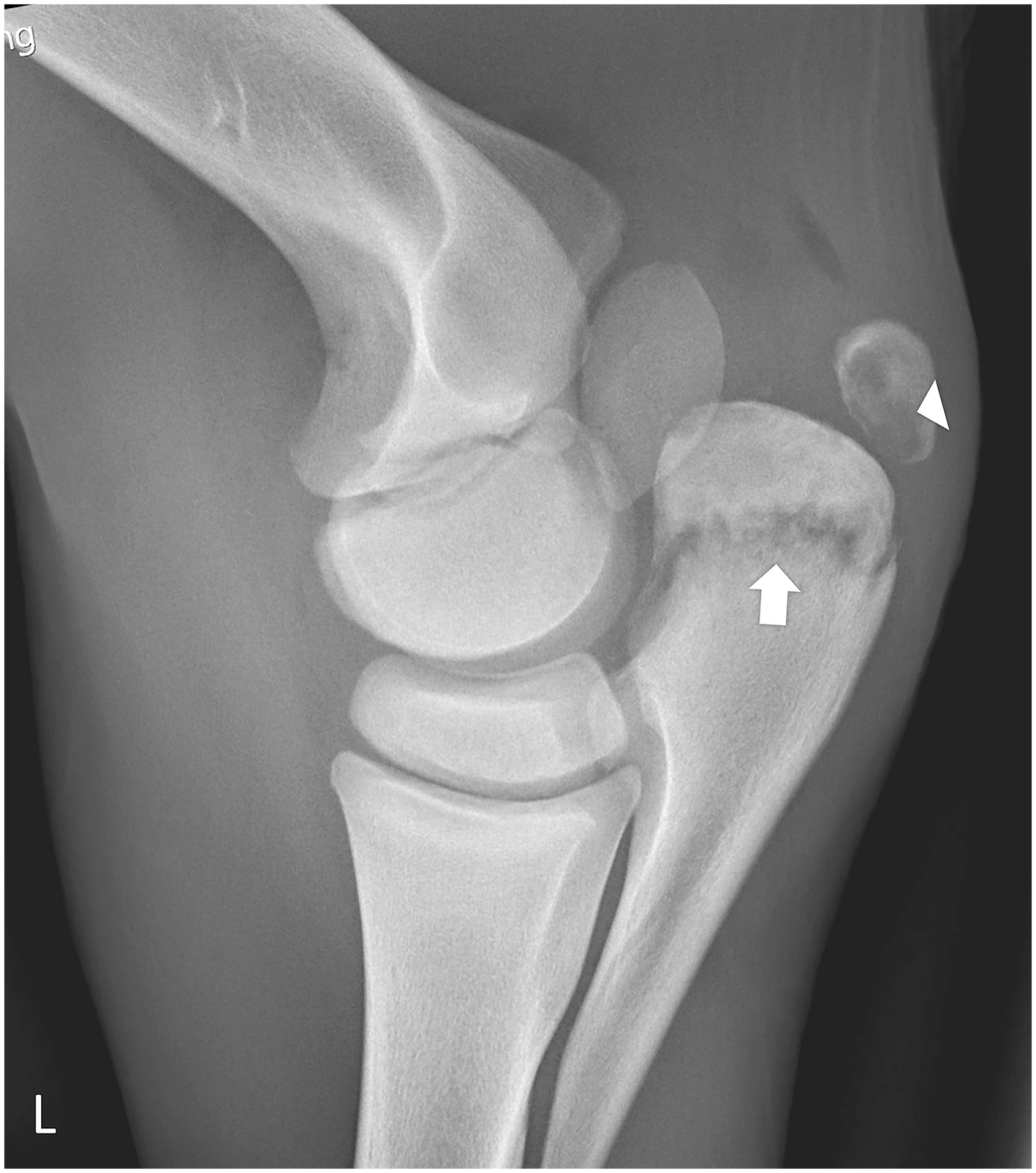
Mediolateral radiographic projection of the left radiohumerocubital joint showing progression of the osteolytic lesions. Radiolucent areas have extended cranially toward the humeroulnar articulation and caudally, resulting in cortical discontinuity (white arrow). The apophyseal lesions have increased in size (white arrowhead), indicating worsening of the osteomyelitic process.

Synovial fluid analysis of the left elbow was performed on days 9, 12, and 14 (Table 1), but no significant improvement in inflammatory parameters was observed. Clinically, the filly’s condition worsened, with progressive left forelimb lameness approaching non–weight-bearing severity (5 out of 5 AAEP scale). Radiographic reassessment revealed progression of osteomyelitis involving the proximal olecranon and adjacent physis, with evidence of communication between the lesion and the joint space (Figure 2).

**Table 1:** Chronological summary of treatments administered and corresponding synovial fluid analysis results for the left radiohumerocubital joint.

Synovial fluid	Day 7	Day 9	Day 10	Day 12	Day 14	Day 17	Day 18	Day 20	Day 22	Day 23	Day 27
TNCC	25,000 cells/μL	66,000 cells/μL		Smear only	81,900 cells/μL			59,800 cells/μL	132,700 cells/μL		
TP	40 g/L	34 g/L			35 g/L			46 g/L	55 g/L		
Neutrophils	88% Non-degenerate	85% Non-degenerative		95% Non-degenerative	93% Mildly lytic			Not assessed Moderately degenerative	>95% Moderate to markedly degenerative		
Treatment	Needle lavage Meropenem 250 mg	Needle lavage Meropenem 500 mg	Needle lavage Meropenem 500 mg CB-MSCs—15 million each site	Needle lavage Meropenem 500 mg CB-MSCs—15 million each site	Joint lavage Meropenem 500 mg CB-MSCs—15 million each site	L elbow joint and partial olecranon arthroscopic debridement, lavage, medication 500 mg Amikacin+ Amikacin beads (fracture site)	Joint medication 500 mg Amikacin	Joint medication 500 mg Amikacin	Joint medication - 500 mg Amikacin		
Other			Gr. 4/5 LF limb lameness Opioids started Pneumonia improving	LF lameness worse Creatinine 166 mmoL/L IV fluids therapy			Gr. 5/5 LF lameness 24 h after the surgery	CB-MSCs—50 million IV Creatinine 111 mmoL/L IV fluid therapy D/C Improved lameness – Gr. 4/5 LF Opioid dose tapered		Discharged Moderate to marked Grade 4/5 LF limb lameness, persistent but slowly improving	CB-MSCs—50 million IV Very mild Grade 4/5 LF limb lameness

The surgical site from the umbilical resection healed without complication, and thoracic ultrasonography indicated improvement in pulmonary pathology consistent with resolving pneumonia. Serum biochemistry, performed to assess renal function, showed an elevated creatinine concentration of 166 μmol/L (reference range: 80–130 μmol/L). Intravenous fluid therapy was initiated using lactated Ringer’s solution at a rate of 4 mL/kg/h, and hydromorphone (0.03 mg/kg IV every 6 h) was added to the analgesic protocol.

Synovial fluid culture from the left elbow yielded *Klebsiella pneumoniae*, which was susceptible to amikacin, ceftiofur, chloramphenicol, enrofloxacin, gentamicin, and tetracycline. Based on antimicrobial susceptibility results, systemic antibiotic therapy was continued with chloramphenicol (50 mg/kg PO q6h).

**Figure 3:**
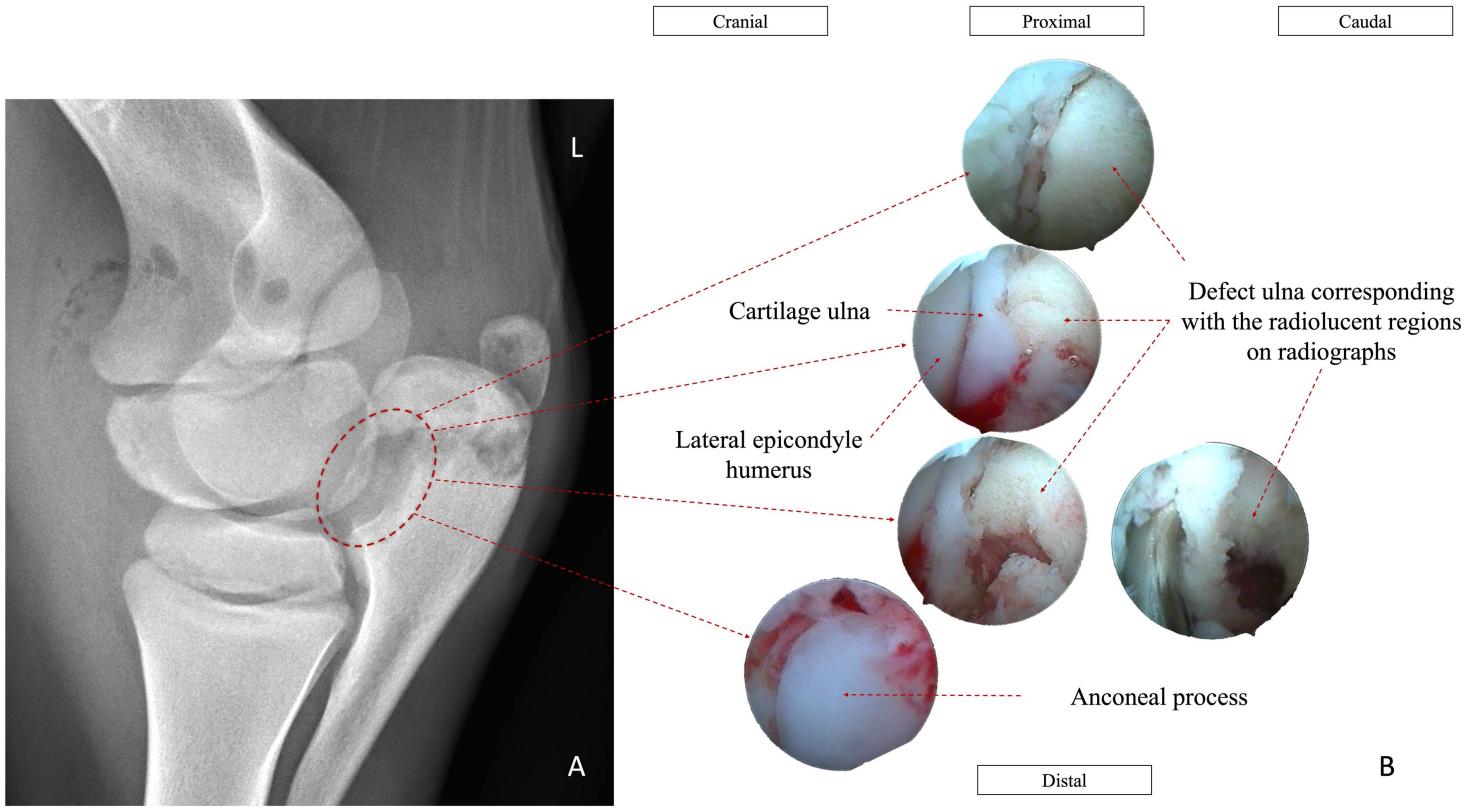
**(A)** Dorsolateral-palmaromedial oblique radiographic view of the left radiohumerocubital joint. **(B)** Arthroscopic image of the caudolateral compartment of the same joint, demonstrating marked fibrin accumulation and synovial inflammation.

On day 17, the owner consented to arthroscopic evaluation of the left elbow joint. Arthroscopy revealed severe synovitis and capsulitis, marked intra-articular fibrin accumulation, and erosion of the articular surface along the lateral aspect of the proximal olecranon. Conservative debridement was performed, taking care to avoid compromising the structurally weakened area associated with the pathological fracture (Figure 3). Fibrin was removed, the joint was thoroughly lavaged, and amikacin (4 mg/kg IA) was administered intra-articularly. In addition, amikacin-impregnated polymethylmethacrylate (PMMA) beads were placed subcutaneously at the caudolateral aspect of the proximal olecranon near the fracture site.

Intra-articular amikacin (4 mg/kg IA) was administered again on days 18, 20, and 22. Despite ongoing therapy, synovial fluid parameters remained unchanged (Table 1). On day 22, a dose of 50 million non-activated CB-MSCs (1 million cells/kg IV) was administered intravenously prepared as previously described (26). Intravenous fluid therapy was discontinued once serum creatinine normalized (111 μmol/L).

Following arthroscopy, the filly showed gradual improvement in comfort and use of the left forelimb, although moderate lameness persisted at the walk (4 out of 5 AAEP scale). Opioid analgesia was discontinued. A second arthroscopic lavage and debridement was recommended; however, the owner declined further surgical intervention and opted to continue treatment at home. The filly was discharged on day 23.

At the time of discharge, moderate lameness was still evident at the walk (4 out of 5 AAEP scale). The filly was prescribed a three-week course of systemic chloramphenicol (50 mg/kg PO q6h), along with a tapering regimen of flunixin meglumine (0.5 mg/kg q12h) and gastroprotective therapy consisting of omeprazole (4 mg/kg PO q24h) and sucralfate (20 mg/kg PO q8h).

On day 27 post-admission, a second intravenous dose of 50 million non-activated CB-MSCs was administered. At that time, the filly showed significant clinical improvement, with only mild lameness observed at the walk (4 out of 5 AAEP scale).

**Figure 4:**
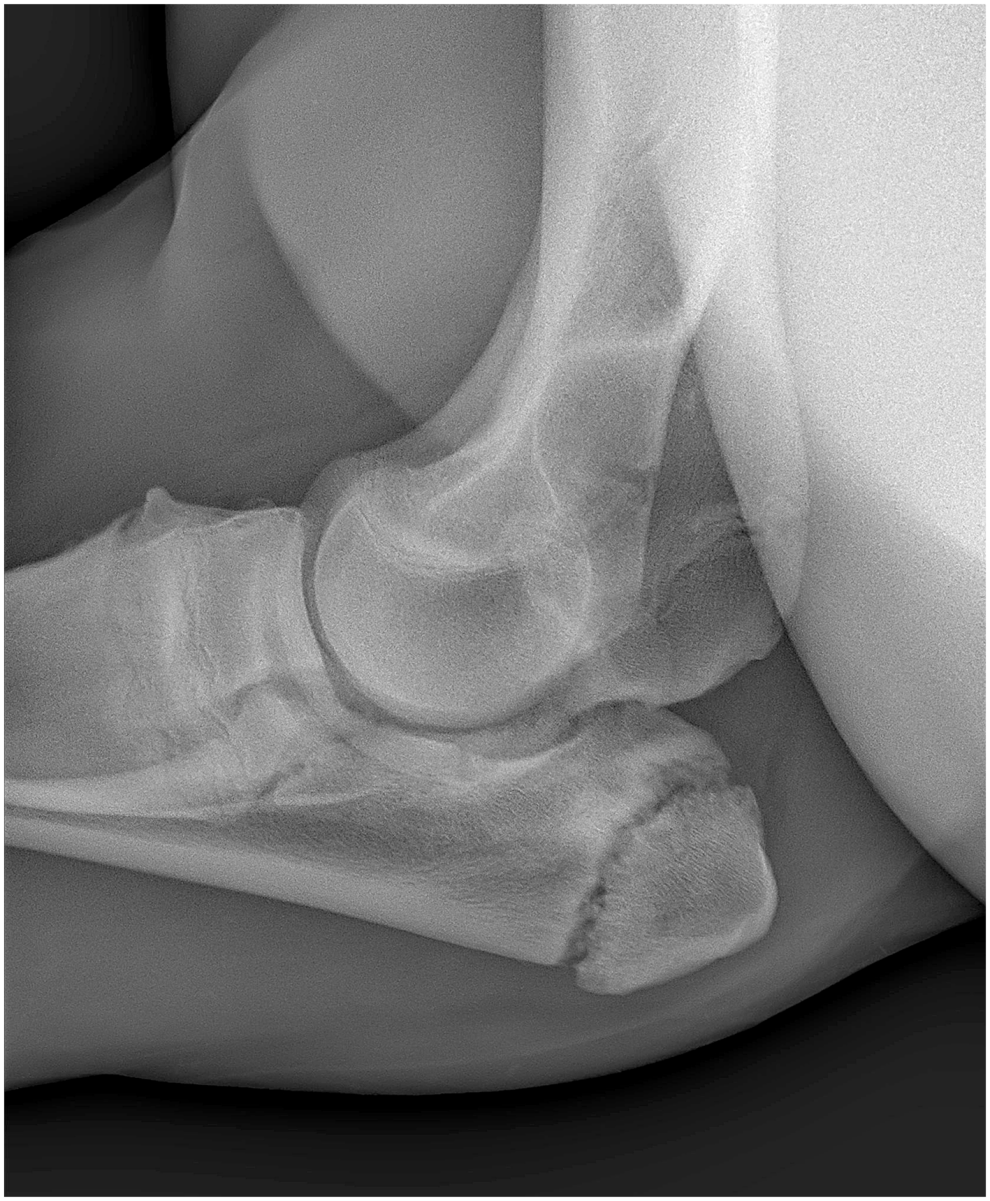
Follow-up mediolateral radiographic projection of the left radiohumeral joint at 13 months post-treatment. The image shows substantial remodeling of the proximal ulna with residual sclerosis of the distal apophysis. No radiographic evidence of osteoarthritic changes is present.

The owner was consistently cooperative and actively engaged in the follow-up process. Monthly clinical evaluations were performed over a 12-month period following discharge, allowing for ongoing monitoring of the foal’s clinical status and long-term outcome. The filly progressively regained full function of the affected limb, with no lameness noted at the walk, trot, or canter. Follow-up radiographs at 12 months (Figure 4) demonstrated substantial remodeling of the proximal ulna, with persistent sclerosis of the distal aspect of the olecranon apophysis. No radiographic evidence of osteoarthritic changes was observed within the joint.

## 3 Discussion

This report highlights the successful use of equine allogeneic umbilical cord-derived mesenchymal stromal cells (CB-MSCs), in combination with systemic and local antimicrobial therapy, to manage a foal with a refractory intra-synovial and osseous septic process.

Numerous studies have demonstrated that MSCs, either directly or through their conditioned media, can inhibit the growth of both Gram-positive and Gram-negative bacteria, including *Staphylococcus aureus*, *Escherichia coli*, *Pseudomonas aeruginosa*, and *Staphylococcus epidermidis* (14–16, 28, 53). Of particular interest in veterinary medicine is the ability of MSCs to destabilize biofilms formed by methicillin-resistant *Staphylococcus aureus* (MRSA) through the secretion of cysteine proteases. This enhances the efficacy of co-administered antibiotics and facilitates bacterial clearance (14, 15, 28). Notably, this anti-biofilm effect has been confirmed both *in vitro* and *in vivo*, including in equine models (14, 29).

TLR3-activated CB-MSCs have shown potential to augment antimicrobial, anti-inflammatory, and immunomodulatory effects. In equine experimental models, this approach, when combined with antibiotics, successfully eliminated MRSA-induced septic arthritis (14). Activated MSCs exert anti-biofilm activity, including against floating biofilm aggregates, and significantly reduce bacterial burden in synovial fluid compared to antibiotic treatment alone. Similar results have been reported in rodent and canine models (14, 20, 21, 23). These findings not only support the use of MSCs for managing drug-resistant infections in veterinary species but also underscore their translational relevance to human medicine, where multidrug-resistant (MDR) infections are increasingly prevalent (20, 30).

Human MSCs may have even stronger antimicrobial effects and more robust responses to immune activation compared to MSCs from other species, suggesting potential for future clinical applications in people with chronic, drug-resistant infections. Several clinical trials are already exploring the use of MSCs in the treatment of acute infections, such as bacterial pneumonia (31).

The demonstrated efficacy of immune-primed MSC therapy in treating MRSA septic arthritis in a large animal model underscores its potential as an innovative treatment for localized orthopedic infections caused by MDR organisms. Importantly, repeated intra-articular MSC injections were well tolerated, supporting their feasibility for routine use in veterinary practice (18).

An especially promising aspect of this approach is its translational potential: horses serve as a valuable large animal model for human joint infections due to similarities in joint anatomy, cartilage thickness, articular loading, and joint volume (32). However, the exact *in vivo* mechanisms by which TLR3-activated MSCs exert therapeutic effects remain incompletely understood and require further investigation (18).

In the present case, the foal developed osteomyelitis of the proximal olecranon involving the apophysis, physis, and metaphysis, complicated by a pathological fracture and secondary septic arthritis of the left radiohumeral (elbow) joint. The lesion was classified as a P-type (physeal) septic arthritis-physitis-osteomyelitis (SAPO) lesion with subsequent joint involvement. Although P-type lesions are more typical in older foals (weeks to months of age) (5, 33, 34), a large retrospective study reported their occurrence in foals younger than previously described (1, 35), suggesting age is not a limiting factor for lesion classification.

Joint involvement is generally associated with a poorer prognosis, particularly with E-type (epiphyseal) lesions. P/E-type combinations carry an elevated risk of poor outcomes for athletic performance, and P-type lesions are more prone to pathological fracture (1, 34). While short-term survival appears unaffected by lesion type, it is influenced by age (≤30 days), the presence of concurrent septic arthritis, and the number of joints affected (1). Ulnar osteomyelitis was rare in one study (4/108 cases), with 3/4 foals surviving short-term and 2/3 returning to race—but notably, none had concurrent pathological fracture or MDR infection (1).

The current gold standard for SAPO management involves rapid diagnosis, systemic broad-spectrum antimicrobials, aggressive surgical debridement of necrotic tissue, and local antimicrobial therapy (3, 5, 6). Prompt intervention is critical, as SAPO is associated with early vascular compromise, ischemia, and bone necrosis (1, 5). Necrotic bone acts as a persistent source of infection, impeding systemic antibiotic penetration and host immune responses (34, 36). Surgical debridement facilitates antimicrobial access, reduces inflammatory burden, and mitigates damage to critical structures such as the physis and joint cartilage (3, 10, 36–38).

In this case, initial management focused on repeated joint lavage with intra-articular and intra-lesional antibiotics and activated CB-MSCs. Owing to rapid lesion progression and limited clinical response, arthroscopic lavage with conservative debridement was subsequently performed. Extensive debridement was avoided because of lesion inaccessibility and concern for joint instability. This approach is consistent with retrospective data indicating that surgical debridement is not uniformly required for a favorable outcome, with only 42 of 108 reported SAPO cases undergoing debridement (1).

Some foals may recover with antimicrobial therapy alone (36). In such cases, resolution may be achieved through host immune responses supported by local and systemic antibiotics, with MSC therapy used adjunctively.

Prompt initiation of antimicrobial therapy is essential. Empirical broad-spectrum antibiotics are typically administered until culture and susceptibility results are available. Although Gram-negative organisms remain common in neonates, an increasing prevalence of Gram-positive infections associated with antimicrobial resistance has been reported (10–12, 17). Careful antibiotic selection is therefore critical. Local antimicrobial delivery is frequently used in conjunction with systemic therapy to achieve high intra-lesional concentrations while minimizing systemic toxicity (8, 38).

The use of critically important antibiotics (CIAs), such as meropenem, should be strictly reserved for life-threatening, culture-confirmed multidrug-resistant infections (2, 3, 8, 39, 40). In this case, meropenem was administered intra-articularly and intra-lesionally because of the foal’s clinical deterioration and lack of response to prior antimicrobial therapy. Although susceptibility data were unavailable at the time of administration, the decision was guided by clinical judgment given treatment failure. Once culture results identified *Klebsiella pneumoniae* susceptible to alternative agents, antimicrobial therapy was transitioned to chloramphenicol. Notably, meropenem was not administered systemically.

Antimicrobial stewardship must remain central to clinical decision-making, particularly in the management of severe infections in neonatal and juvenile animals. Although meropenem is not appropriate for routine first-line therapy, it may be considered in exceptional circumstances involving a life-threatening infection, suspected or confirmed multidrug-resistant organisms, or failure of standard antimicrobial regimens. However, the pharmacokinetics, pharmacodynamics, safety profile, and optimal dosing strategies of meropenem in veterinary species—particularly in foals—remain poorly defined. Prospective, controlled studies are therefore needed to evaluate safety, clinical utility, and potential impacts on antimicrobial resistance, and to inform evidence-based guidelines supporting judicious use in veterinary medicine (40, 41).

Additionally, antibiotic selection should consider potential interactions with MSCs. Some antimicrobials negatively affect MSC viability, gene expression, and differentiation capacity (42, 54). Aminoglycosides at high concentrations reduce total RNA expression and suppress osteogenesis/chondrogenesis in equine and human MSCs (43–45). Cephalosporins and fluoroquinolones also negatively impact MSC viability and function (45, 46). In contrast, tetracyclines such as doxycycline and minocycline have been shown to enhance MSC chondrogenesis and immunomodulation (47–49).

In this case, systemic MSC administration was initiated after local treatment was changed to amikacin, to avoid potential local cytotoxic effects. Currently, there are no evidence-based dosing recommendations for MSC administration in foals with infection. For musculoskeletal injuries, some protocols suggest delayed administration (20–30 days post-injury), with high doses (>20 million MSCs) and repeated treatments spaced 2–4 weeks apart (50, 51). The most commonly reported systemic dose in humans and rodents is 1 million MSCs per kg body weight (52). However, dosing regimens for systemic or intra-lesional MSC therapy in equine infectious disease remain undefined.

In this case, treatment frequency and dosage were determined in consultation with the originating research group and do not represent established or evidence-based clinical standards.

Despite the complexity of this case and the presence of multiple complicating factors—including a pathological fracture, a multidrug-resistant infection, and a lack of response to conventional therapy—the combined use of antimicrobial treatment and MSC therapy led to a favorable outcome. This case adds to the growing body of evidence suggesting that MSCs, particularly when immune-primed, may serve as a valuable adjunct in the management of refractory or multidrug-resistant infections.

Interpretation of treatment response is limited by therapeutic confounding, as multiple interventions were administered concurrently and sequentially, precluding isolation of the specific contribution of MSC therapy to the clinical outcome. Synovial fluid analyses revealed persistently elevated total nucleated cell counts with progressive neutrophil degeneration throughout much of the treatment period, suggesting an ongoing intra-articular septic process. Consequently, the apparent clinical resolution is difficult to reconcile with the initial disease progression and the lack of an early, measurable response to therapy. Delayed clinical improvement cannot be excluded; however, additional follow-up synovial evaluations were not performed.

Further research is urgently needed to: (1) Determine optimal MSC dosing, timing, and delivery strategies; (2) Compare immune-primed versus quiescent MSCs *in vivo*; (3) Evaluate MSC-antibiotic interactions across antimicrobial classes; (4) Assess long-term outcomes and safety of MSC therapy in joint and bone infections.

MSC therapy represents a novel and promising strategy for managing multidrug-resistant orthopedic infections in equine patients—including septic arthritis, osteomyelitis, and distal limb wounds—and may also hold translational value for human medicine.

## Data Availability

The raw data supporting the conclusions of this article will be made available by the authors, without undue reservation.
